# Hypnotism

**Published:** 1860-10

**Authors:** 


*Hypnotism.*—Our English exchanges and some French journals are being troubled about hypnotism. It is nothing more than a new form of mesmerism, only more of a shadow. That fixity of vision may produce many mental phenomena, we doubt not, but that it can overcome the greatest law of the animal economy, the most powerful instinct, we think most unreasonable.

It is useless to attempt to foist upon the intelligent of this day any nonsense, especially unsupported by a rational hypothesis. This hypnotism is a naked foundling, which the proper authorities will no doubt trace to the parentage of clairvoyance. B.

